# Synergistic Antibacterial Activity of Basil and Ginger Essential Oils and Their Preservative Effect on Braised Beef

**DOI:** 10.3390/foods15010122

**Published:** 2026-01-01

**Authors:** Yunshuang Man, Rongrong Yang, Weijing Xu, Ye Liu, Yinying Luo, Lin Mei, Jun Qi, Lele Shao

**Affiliations:** Key Laboratory of Jianghuai Agricultural Product Fine Processing and Resource Utilization, Ministry of Agriculture and Rural Affairs, Anhui Engineering Research Center for High Value Utilization of Characteristic Agricultural Products, School of Food and Nutrition, Anhui Agricultural University, Hefei 230036, China

**Keywords:** fractional inhibitory concentration, antibacterial mechanism, meat product, shelf life

## Abstract

Plant essential oils have gained attention for their green and safe characteristics in recent years. However, negative effects on sensory attributes caused by high concentrations hinder their application in foods. The synergistic antibacterial activity and mechanism of basil (BEO) and ginger (GEO) essential oils against *Escherichia coli* and *Staphylococcus aureus* were investigated in this study. The preservative effect on braised beef, a Chinese traditional meat product, of combined BEO and GEO was also studied. Both BEO and GEO displayed notable antibacterial activity when applied individually against *E. coli* and *S. aureus*. Moreover, the combination of BEO and GEO exhibited synergistic activity, with a fractional inhibitory concentration index of 0.75. The BEO + GEO combination reduced bacterial metabolism, ruptured bacterial membranes, reduced membrane potential, and destructed intracellular enzymes and the membrane integrity of *E. coli* and *S. aureus*. The application of BEO + GEO in braised beef could effectively maintain its quality and prolong its shelf life by inhibiting bacterial growth, preventing texture changes and color deterioration. The combination of BEO and GEO exhibited a synergistic antibacterial activity, providing effective preservation of braised beef. The findings contribute valuable insights into the development of natural antibacterial preservatives for meat products.

## 1. Introduction

Braised beef is one of the typical seasoned meat products in China and many other Asian countries, which is broadly loved by consumers. The dish is produced by stewing beef with condiments to further enhance the flavor of the beef [1]. However, braised beef, like other meat products, is particularly susceptible to spoilage during processing, transportation, and preservation [2]. Braised beef is basically preserved at room temperature and 4 °C in the Chinese market, which results in a large amount of braised beef being wasted because it cannot be consumed within a short period of time [3]. Food contamination includes not only spoilage bacteria but also foodborne pathogens, which are a serious threat to public health [2]. Therefore, prolonging shelf life by controlling and diminishing microbial contamination is the primary bottleneck for the food industry.

Synthetic chemical agents are traditionally employed to prevent bacterial growth in the food industry. However, consumer demand for minimally processed foods is escalating, prompting the development of natural-based approaches to replace synthetic additives [4]. As an alternative to synthetic additives, essential oils (EOs) are complex mixtures of secondary metabolites commonly acknowledged as natural antimicrobial agents [5]. Recently, EOs have demonstrated significant antimicrobial and preservative effects in a variety of food products, such as snakehead fish [6], beef jerky [7], and pork [8]. Ceylan et al. [9] reported that curcumin and rosemary EOs extended the shelf life of fish fillets from 6 days to 27 days at 4 °C by reducing microbial spoilage. Lotfy et al. [10] also found that *Citrus limon* EO and its nanoemulsion could effectively inhibit the growth of *Escherichia coli* in minced beef during refrigerated storage, extending the shelf life. However, the usage of EOs is limited in foods, mainly as a result of the high concentrations required to inhibit the growth of microbial cells, which could cause negative effects on sensory attributes, including alterations in color, odor, and texture [5].

The utilization of the synergistic antibacterial activity of various EOs is one of the efficient approaches to tackle the issue of EOs. The synergistic interaction among EOs manifests as higher antibacterial efficacy exceeding the sum of their individual activities. Consequently, combinations of various EOs possessing synergistic antibacterial activity can reduce the required concentrations of the substances [5]. He et al. [5] observed that thyme and clove EOs possessed a synergistic effect against *Malassezia furfur*. Kim et al. [11] also reported that cinnamon bark and thyme thymol EOs showed synergistic activity against *L. monocytogenes* in tomato juice at 10 °C and 25 °C for up to 48 h. However, the antimicrobial mechanism of EO combinations and their preservative effects on food need further elucidation.

Basil (*Ocimum basilicum* L.) is a herb belonging to the *Lamiaceae* family, considered the most cultivated aromatic herb variety worldwide. It has been reported that basil essential oil (BEO) possesses antimicrobial, antifungal, antioxidant, and anti-inflammatory activities [12]. Ginger essential oil (GEO) is the volatile oil extracted from the root of ginger. Due to its unique fragrance and biological activity, it has very broad development prospects in the pharmaceutical, food, and cosmetics industries [13]. GEO has been extensively investigated with a special focus on its antioxidant, antifungal, and antibacterial activities [14]. Therefore, in this study, the antibacterial effects of BEO and GEO when used individually against *E. coli* and *Staphylococcus aureus* were investigated. Meanwhile, the synergistic antibacterial activity of BEO + GEO was also determined with the fractional inhibitory concentration index (FICI) and time-killing assay. The synergistic mechanism of BEO and GEO against *E. coli* and *S. aureus* was also elucidated by laser scanning confocal microscopic observation and determination of bacterial metabolism, membrane potential, and integrity. In addition, braised beef, one of the traditional Chinese meat products, was employed to study the preservative effect of combined BEO and GEO by determining the changes in total viable count (TVC), texture profile, color, and pH.

## 2. Materials and Methods

### 2.1. Chemicals and Bacterial Strains

BEO (eugenol > 85%, linalool 10–15%) and resazurin sodium salt were obtained from Shanghai Yuanye Biotechnology Co., Ltd., Shanghai, China. GEO (gingerol ≥ 35%) was obtained from Shandong Suihua Biotechnology Co., Ltd., Jinan, China. Propidium iodide (PI) and 5(6)-carboxyfluorescein diacetate (cFDA) were obtained from Shanghai Aladdin Biochemical Technology Co., Ltd., Shanghai, China. Beef shank, salt, sugar, ginger, and other seasonings (star anise, fennel, chili, Tsaoko, Sichuan pepper, and soybean sauce) were purchased from a local market in Hefei, China. Nutrient agar (NA) and nutrient broth (NB) were purchased from Hangzhou Baisi Biotechnology Co., Ltd., Hangzhou, China. *Escherichia coli* ATCC 25922 and *Staphylococcus aureus* ATCC 25923 were employed in this study. The strains were separately subcultured onto NA and then incubated at 37 °C for 24 h. After that, a single colony was mixed with 20 mL of fresh NB, and then the suspension was incubated for 12 h at 37 °C prior to use.

### 2.2. The Antibacterial Activities of BEO and GEO

#### 2.2.1. Determination of Minimum Inhibitory Concentrations (MICs)

The MICs of BEO and GEO against *E. coli* and *S. aureus* were determined via the serial dilution method. Serial dilutions of BEO mixture (0.31 to 20.00 mg/mL) were conducted in a sterile 96-well plate using NB. A quantity of 20 μL of the bacterial suspension (~10^6^ CFU/mL) was added to each of the wells. Next, the mixtures were incubated at 37 °C for 24 h after being shaken slightly. The absorbance values at 600 nm were recorded using a microplate reader (SpectraMaxM2e; Molecular Devices, San Jose, CA, USA). NB medium added with or without bacteria added were also carried out as controls.

#### 2.2.2. The Determination of FICI

The potential synergistic effect of BEO + GEO was evaluated using the FICI, which was determined according to the method of Fratini et al. [15]. Six concentrations of BEO and GEO were prepared (8 MIC, 4 MIC, 2 MIC, MIC, 1/2 MIC, and 1/4 MIC). BEO dilutions (100 μL) were applied along the X-axis of a 96-well microplate arranged in a checkerboard pattern, with GEO dilutions distributed along the Y-axis in order to obtain the final concentrations (4 MIC, 2 MIC, MIC, 1/2 MIC, 1/4 MIC, and 1/8 MIC). A quantity of 20 μL of bacterial suspension (*E. coli* or *S. aureus*, ~10^6^ CFU/mL) was added. After incubation at 37 °C for 24 h, the absorbance values at 600 nm were recorded. NB with or without bacteria were also carried out as controls. The FICI was calculated using Equation (1). An FICI value < 1 is considered to indicate a synergistic effect; an FICI value = 1 means a commutative effect; 1 < FICI value ≤ 2 represents an indifferent effect; and an FICI value > 2 indicates an antagonistic effect.

(1) $$FICI=\frac{C_{BEO}}{{MIC}_{BEO}}+\frac{C_{GEO}}{{MIC}_{GEO}}$$

where *C*_BEO_ is the MIC of BEO in combination with GEO and *C*_GEO_ is the MIC of GEO in combination with BEO.

#### 2.2.3. Time-Killing Analysis of BEO and GEO

Time-killing assays of GEO and BEO against *E. coli* and *S. aureus* were conducted, including GEO (MIC), GEO (1/4 MIC), BEO (MIC), BEO (1/2 MIC), and GEO (1/4 MIC) + BEO (1/2 MIC). A quantity of 200 μL of EO solution was added into a 96-well plate, then 20 μL bacterial suspension (*E. coli* or *S. aureus*) was added to obtain a concentration of 1 × 10^5^ CFU/mL. The suspensions were incubated at 37 °C, and the optical density of the bacterial suspensions was determined at 600 nm using the microplate reader after 0, 2, 4, 6, 12, and 24 h.

### 2.3. The Antibacterial Mechanism of BEO and GEO

#### 2.3.1. Assessment of Membrane Integrity

The membrane integrity of *E. coli* and *S. aureus* was determined using the PI staining assay [16]. Initially, a stock solution of PI (1.5 mM) was prepared by dissolving it in deionized water and then storing it in the dark at 4 °C. The bacterial suspension was inoculated with GEO (MIC), GEO (1/4MIC), BEO (MIC), BEO (1/2 MIC), and GEO (1/4MIC) + BEO (1/2 MIC) separately at 37 °C for 4 h. Then, 1 mL of PI stock solution was added into the mixture. After incubation for 30 min at 37 °C, the fluorescence intensities (Ex at 530 nm and Em at 590 nm) were detected using the microplate reader. *E. coli* and *S. aureus* suspensions without EO solutions were also performed as controls.

#### 2.3.2. Determination of Membrane Potential

The membrane potential of *E. coli* and *S. aureus* cells was investigated via the JC-1 assay [17]. In brief, a bacterial suspension (~10^6^ CFU/mL) was mixed with GEO (MIC), GEO (1/4 MIC), BEO (MIC), BEO (1/2 MIC), and GEO (1/4 MIC) + BEO (1/2 MIC) separately. The mixtures were centrifuged at 8000× *g* for 10 min (4 °C) after incubation for 4 h and subsequently resuspended in sterile saline (0.9% NaCl). To each mixture, JC-1 solution was added to a final concentration of 2 μM, followed by incubation for 30 min at 37 °C in the dark. The excess JC-1 was washed away using sterile saline by centrifugation at 8000× *g* for 10 min. The red (Ex 585 nm and Em 590 nm) and green (Ex 515 nm and Em 529 nm) fluorescence intensities of each treatment were determined using the microplate reader.

#### 2.3.3. Measurement of Intracellular Enzyme Activity

The intracellular enzyme (esterase) activity of *E. coli* and *S. aureus* exposed to EOs was determined using cFDA [16]. After being treated with GEO (MIC), GEO (1/4 MIC), BEO (MIC), BEO (1/2 MIC), and GEO (1/4 MIC) + BEO (1/2 MIC) separately for 4 h, cFDA was added to the bacterial suspensions. Then the mixture was incubated at 37 °C for 30 min. The fluorescence intensity with Ex 485 nm and Em 528 nm was recorded with the microplate reader. Untreated bacterial suspensions were also performedas controls.

#### 2.3.4. Measurement of Bacterial Metabolism

The bacterial metabolism of *E. coli* and *S. aureus* exposed to GEO and BEO was determined with the Resazurin assay [18]. Briefly, EO suspensions (200 μL), including a control (no addition), GEO (MIC), GEO (1/4 MIC), BEO (MIC), BEO (1/2 MIC), and GEO (1/4 MIC) + BEO (1/2 MIC), were added to a sterile 96-well plate, then 20 μL of bacterial suspension (~10^6^ CFU/mL) was added to each of the wells. After incubation at 37 °C (4 h), 30 μL of resazurin sodium salt solution (100 μg/mL) was dispensed into each well. The mixtures were incubated at 37 °C for 2 h away from light. The fluorescence intensity was subsequently tested using the microplate reader with Ex 530 nm and Em 590 nm.

#### 2.3.5. Laser Scanning Confocal Microscopic Observation

Laser scanning confocal microscopy was utilized to further evaluate the membrane integrity and intracellular enzyme activity of bacterial cells subjected to GEO and BEO. After being exposed to GEO (MIC), GEO (1/4 MIC), BEO (MIC), BEO (1/2 MIC), and GEO (1/4 MIC) + BEO (1/2 MIC) separately for 4 h at 37 °C, PI and cFDA solutions were successively added to the bacterial suspensions to obtain final concentrations of 1.5 mM and 1 mM, respectively. Subsequently, the mixtures were incubated at 37 °C for 30 min. Then, the bacterial suspensions were dripped onto slides and covered with cover slips. Fluorescence microscopic images were acquired using the Stellaris 5 confocal laser microscope (Leica Microsystems, LAS X 4.7). The excitation wavelength was set as 488 nm, and the emission wavelengths at 528 nm and 638 nm were chosen for cF and PI, respectively. Untreated *E. coli* and *S. aureus* suspensions were also performed as controls.

### 2.4. The Preservative Effect of BEO + GEO on Braised Beef

#### 2.4.1. The Preparation of Braised Beef

**Figure 1:**
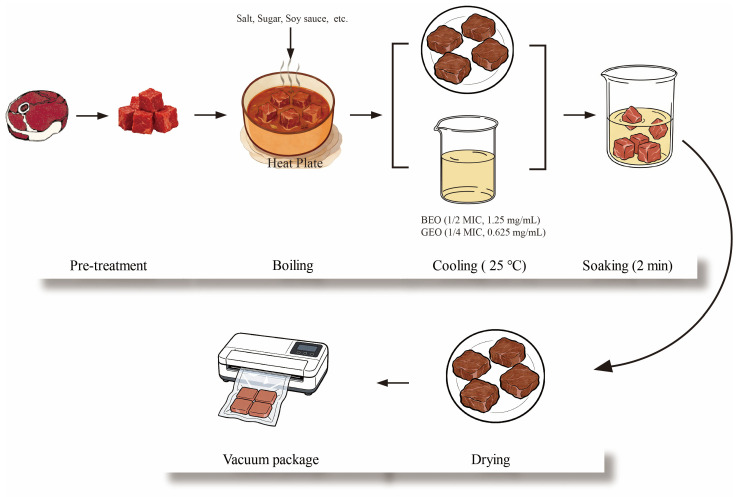
Diagram of braised beef production.

The preparation of the braised beef is shown in Figure 1. Briefly, the beef shank was cut into cubes (about 200 g), which were then pre-cooked in a water bath from 25 to 100 °C. After that, the beef meat cubes were immersed in seasoned broth. The seasoned broth was prepared by stewing a mixture containing water (10 kg), soybean sauce (0.4 kg), salt (0.4 kg), sugar (0.2 kg), ginger (200 g), fennel (50 g), Sichuan pepper (200 g), capsicum (50 g), cinnamon (50 g), and star anise (20 g). Next, the mixture was boiled for 10 min and simmered for 1 h. Subsequently, the resulting beef was cooled to room temperature. Based on the antibacterial synergistic effect, the obtained braised beef was dipped in the mixed solution of BEO (1/2 MIC, 1.25 mg/mL) and GEO (1/4 MIC, 0.625 mg/mL) for 2 min; an obviously unpleasant odor was not smelled at such concentrations. Another group of beef samples dipped in sterile water were performed as negative controls. The treated beef was then dried in a biosafety cabinet and packaged using a vacuum packaging machine (ZK013; KONKA Group Co., Ltd., Shenzhen, China). Three independent replicates were performed in this study. The TVC, texture profile, color, and pH of the beef samples were measured at 2-day intervals during storage at 4 °C.

#### 2.4.2. Determination of Total Viable Count

The plate count method was employed to determine the TVC of braised beef during storage. Briefly, beef flesh (5 g) was mixed with 45 mL of sterile saline and then shaken for 30 min, after which the mixture was further diluted to appropriate concentrations using sterile saline. Next, 100 μL of undiluted or diluted mixture was plated on an NA plate, and the counts were enumerated after incubation at 37 °C for 24–48 h. Each sample was conducted in triplicate.

#### 2.4.3. Measurement of pH

The pH of the braised beef was evaluated according to the report of Lan et al. [19]. In brief, a 4 g beef sample was added to 40 mL of potassium chloride solution (pH 7.0, 0.1 M), then the mixture was homogenized (XHF-DY; Ningbo Scientz Biotechnology Inc., Ningbo, China) for 1 min (8000× *g*, 25 °C). The pH of the mixture was tested using a digital pH meter (PHS-3C; INESA Scientific Instrument Co., Ltd., Shanghai, China).

#### 2.4.4. Texture Profile Analysis

A texture analyzer (TA-XT Plus; Stable Micro System, Surrey, UK) with a P50/R probe was employed to determine the texture of the beef samples [20]. The beef samples were cut into cubes (2 × 2 × 2 cm^3^) before testing. The degree of compression and trigger force were 40% and 5.0 g, respectively. Hardness, resilience, and cohesiveness were recorded.

#### 2.4.5. Determination of Color

The color changes in the braised beef during storage at 4 °C were assessed using a CR-400 Minolta chronometer (Konica Minolta, Osaka, Japan). The assay was performed in quadruplicate by random sampling, and L*, a*, and b* were recorded for each sample. The color changes (△*E*) of the braised beef during storage were also calculated using Equation (2):

(2) $$∆E=\sqrt{{\left({∆L}^{*}\right)}^{2}+{\left({∆a}^{*}\right)}^{2}+{\left({∆b}^{*}\right)}^{2}}$$

### 2.5. Statistical Analysis

Data were expressed as means ± standard deviations, with three replicates, in this study. SPSS 27.0 (SPSS Inc., Chicago, IL, USA) was employed for statistical analysis using one-way ANOVA and Turkey’s HSD test, and *p* < 0.05 was considered statistically significant. Figures were drawn using Origin 9.0 (OriginLab, Northampton, MA, USA).

## 3. Results and Discussion

### 3.1. The Antibacterial Activities of BEO and GEO

The MICs of BEO against *E. coli* and *S. aureus*, measured using the serial dilution method in this study, were both 2.5 mg/mL. Stojanovic-Radic et al. [21] also reported that BEO against *Salmonella* Enteritidis showed efficiency at a concentration of 2.5 mg/mL. Previous reports have shown that the MICs of BEO against *S. aureus*, *Serratia marcescens*, and *E. coli* vary from 0.25 to 1.00 mg/g [22]. In terms of GEO, the MICs against *E. coli* and *S. aureus* were both 2.5 mg/mL. Wang et al. [14] also investigated the antibacterial activities of GEO and obtained MICs of 1.0 and 2.0 mg/mL for *S. aureus* and *E. coli*, respectively. The antibacterial ability of EOs depends on chemical composition; the different antibacterial performances of EOs in our study and previous reports might be attributed to their various sources, which could have caused differences in the constituents.

In order to quantitatively evaluate the antagonistic/synergistic effects of combined BEO and GEO on *E. coli* and *S. aureus*, FICIs were calculated for the tested combination. A combination of BEO + GEO showed synergistic effects (FICI = 0.75) against *E. coli* and *S. aureus* at levels of 1/2 MIC and 1/4 MIC, respectively. The results demonstrated that the BEO and GEO combination achieved comparable antibacterial efficacy at ~75% of the total concentration required for treatment with individual EOs. Sethunga et al. [23] also found that GEO + cinnamon bark oil (CBO) exerted a synergistic effect against *Bacillus subtilis*, with an FICI of 0.35. The combination of CBO and thyme thymol oil also exhibited a synergistic inhibitory effect (FICI = 0.5) against *L. monocytogenes*, while thyme thymol oil and oregano oil had an indifferent effect [11]. Burt et al. [24] speculated that both minor and major components of EOs might exert an impact on the interactions that produce synergism. Instead of full synergistic effects, EOs with similar compositions and structures generally display additive or partially synergistic effects [5]. Behbahani et al. [25] suggested that the synergistic effect of two EOs against bacteria might be due to (i) the antibacterial mechanisms of EOs being dissimilar, targeting different aspects of bacteria, or (ii) the synergistic action that results from their mechanisms being similar.

**Figure 2:**
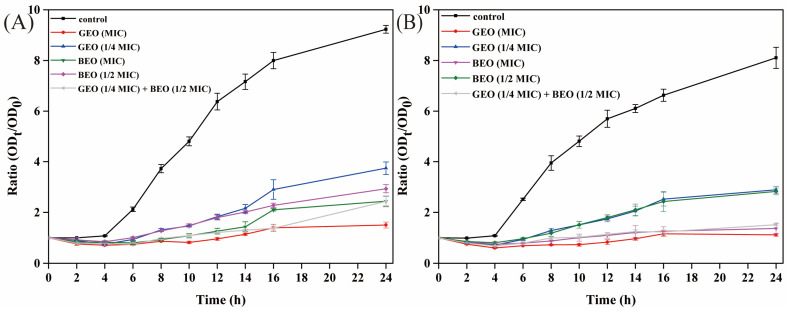
The time-killing curves of BEO, GEO, and their combinations against *E. coli* and *S. aureus*: (**A**) *E. coli*; (**B**) *S. aureus*.

In addition, the antibacterial activities of BEO, GEO, and their combination against *E. coli* and *S. aureus* were assessed by time-killing curves (Figure 2). The results showed that the growth of untreated *E. coli* and *S. aureus* basically followed the model’s shaped growth curve, while the absorbance values of the EO-treated groups were all significantly lower than those of the control groups, which suggested that the EOs inhibited the growth and reproduction of bacteria. The absorbance values of the 1/4 MIC GEO treatment were larger than those of the MIC GEO group; the growth of *E. coli* and *S. aureus* was also significantly inhibited when they were treated with BEO at the MIC compared to the treatment with BEO at 1/2 MIC, indicating that the EO concentration also affected the normal proliferation of bacteria. Additionally, the inhibition effects of the GEO (1/4 MIC) + BEO (1/2 MIC) treatment were similar to those of the treatments of BEO (MIC) and GEO (MIC) alone. The results also confirmed that there was a synergistic antibacterial activity of the combination of BEO and GEO. Furthermore, the utilization of GEO + BEO could reduce the quantity of a single EO, thereby diminishing its sensory odor.

### 3.2. The Antibacterial Mechanism of BEO and GEO in E. coli and S. aureus

#### 3.2.1. Effects of BEO and GEO on Membrane Integrity and Potential

**Figure 3:**
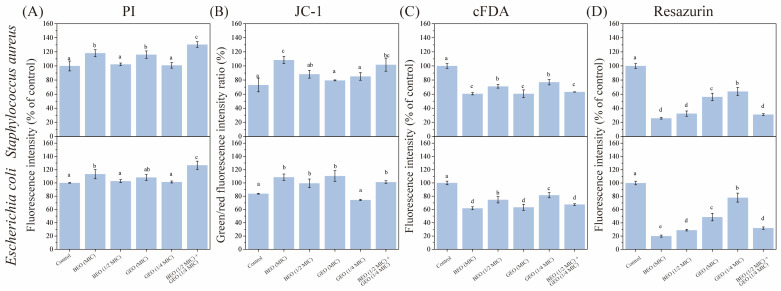
Effects of BEO, GEO, and their combinations on membrane potential (**A**), membrane integrity (**B**), intracellular enzyme activity (**C**), and cell metabolism (**D**) of *E. coli* and *S. aureus*. Note: Different letters (a–e) in the figure indicate significant differences (*p* < 0.05).

The membrane integrity of *E. coli* and *S. aureus* after exposure to BEO and GEO was evaluated through quantitative PI fluorescence intensity analysis, as depicted in Figure 3A. Compared to the control group, all treatments showed higher fluorescence intensities, suggesting that the addition of EOs ruptured the cell membranes of *E. coli* and *S. aureus*. The GEO (1/4 MIC) + BEO (1/2 MIC) group exhibited the highest fluorescence intensity (*p* < 0.05). The main composition of BEO includes 1,8-cineol, linalool, α-bergamotene, 6-methoxyphenol, and γ-cadinene; these compounds have been proven to interact with bacterial cell membranes [26]. In addition, zingiberene, α-farnesene, 6-gingerol, and α-curcumen are the active substances of GEO that contribute to its antibacterial activity, which could affect cell permeability by attacking the cell wall and cytoplasmic membrane [14]. The similar antibacterial mechanisms of GEO and BEO may explain why the GEO (1/4 MIC) + BEO (1/2 MIC) treatment caused the highest degree of membrane damage in this study, their similarity contributing greatly to the synergistic antibacterial activity.

Membrane potential can be recorded as the intracellular–extracellular electrical potential difference [27]. Being a key role of the proton motive force, it powers ATP biosynthesis and impacts cell metabolic activity [27]. In this study, compared to untreated bacterial cells, the membrane potentials of both *E. coli* and *S. aureus* declined after treatment with BEO and GEO (Figure 3B). Moreover, the GEO (1/4 MIC) + BEO (1/2 MIC) and BEO (MIC) treatments were similar with respect to membrane potential. The results were in agreement with the antibacterial activity assays. The depolarization of the cell membranes of *E. coli* and *Burkholderia glumae* after exposure to BEO [28] and GEO [29], respectively, have also been reported previously.

#### 3.2.2. Effects of BEO and GEO on Intracellular Enzyme Activity

With the purpose of quantitatively assessing the changes in intracellular esterase activity, *E. coli* and *S. aureus* subjected to the combination of BEO and GEO for 4 h were incubated with the fluorescent dye cFDA (Figure 3C). cFDA dye can freely penetrate into bacterial cells and is converted by nonspecific esterases into carboxyfluorescein (cF) [30]. Thus, cF fluorescence levels can simultaneously reflect intracellular esterase activity and cellular metabolic activity. Compared to the control group, the fluorescence intensities of all EO-treated *E. coli* and *S. aureus* declined (*p* < 0.05), which indicated that both GEO and BEO could partially destruct intracellular esterase. Furthermore, there were no significant differences in fluorescence intensity among the GEO (1/4 MIC) + BEO (1/2 MIC), BEO (MIC), and GEO (MIC) groups (*p* > 0.05). This result manifested that the combination of BEO and GEO showed synergistic inhibition of esterase activity.

#### 3.2.3. Effects of BEO and GEO on Bacterial Metabolism

Resazurin, a redox indicator, can penetrate bacteria and then produce intermediates with a high fluorescence intensity catalyzed by different oxidoreductases in bacteria [18]. A direct correlation exists between the magnitude of this conversion and the number of metabolically active bacteria. As shown in Figure 3D, all EO treatments resulted in a reduction in the metabolic activity of *E. coli* and *S. aureus* (*p* < 0.05). BEO significantly impacted bacterial metabolism than that of GEO (*p* < 0.05), and GEO (1/4 MIC) + BEO (1/2 MIC) had a similar performance to that of BEO (1/2 MIC) (*p* > 0.05). The findings illustrated that the inhibition activity of GEO + BEO in relation to bacterial metabolism was mainly contributed by GEO. The result was consistent with those of Li et al. [26], who observed that BEO could inhibit the respiratory metabolism of *L. monocytogenes* by producing reactive oxygen species.

#### 3.2.4. Laser Scanning Confocal Microscopy Analysis of *E. coli* and *S. aureus*

**Figure 4:**
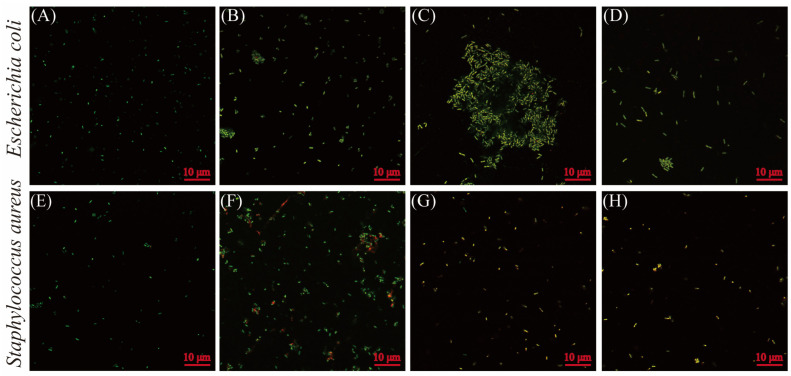
Fluorescence microscopic images of *E. coli* (**A**–**D**) and *S. aureus* (**E**–**H**) subjected to BEO, GEO, and their combination: (**A**) control; (**B**) GEO (1/4 MIC); (**C**) BEO (1/2 MIC); (**D**) BEO (1/2 MIC) + GEO (1/4 MIC); (**E**) control; (**F**) GEO (1/4 MIC); (**G**) BEO (1/2 MIC); (**H**) BEO (1/2 MIC) + GEO (1/4 MIC).

Laser confocal microscopic images of *E*. *coli* and *S*. *aureus* cells subjected to BEO and GEO are shown in Figure 4. PI and cFDA were employed to visually assess cell membrane integrity and esterase activity. Bacterial cells with green fluorescence (cF) represent active esterase activity, whereas red fluorescence (PI) suggests disrupted membranes. Untreated *E. coli* and *S. aureus* exhibited green fluorescence (Figure 4A,E), indicating intact cell membranes and normal enzyme function. Parts of bacterial cells subjected to GEO (1/4 MIC) and BEO (1/2 MIC) alone were stained with red fluorescence, indicating membrane damage in *E. coli* and *S. aureus*. Meanwhile, the GEO (1/4 MIC) and BEO (1/2 MIC) combination treatment resulted in a concurrent reduction in green fluorescence and an elevation in red fluorescence, indicating increased bacterial cell membrane damage and impaired esterase activity. The results were consistent with the findings on membrane integrity and the intracellular enzyme activity assays.

### 3.3. The Preservative Effect of BEO and GEO on Braised Beef

#### 3.3.1. Total Viable Count Changes

**Figure 5:**
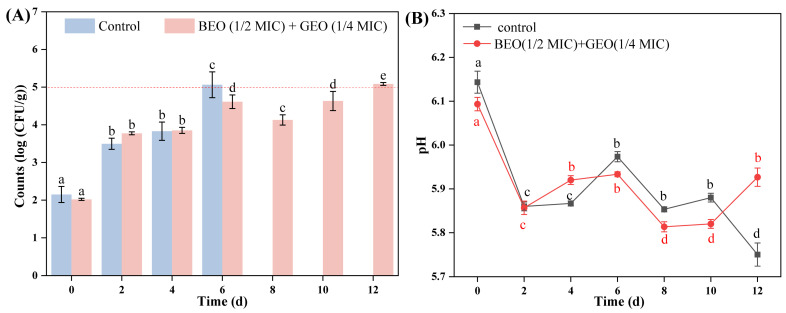
Changes in total viable count (**A**) and pH (**B**) of braised beef treated with BEO (1/2 MIC) + GEO (1/4 MIC) during storage at 4 °C. Note: Different letters (a–e) in the figure indicate significant differences (*p* < 0.05).

The changes in TVC in braised beef treated with BEO (1/2 MIC) + GEO (1/4 MIC) during storage at 4 °C are shown in Figure 5A. The initial TVC (0 d) of untreated and treated braised beef was 2.15 and 2.02 log CFU/g (*p* > 0.05), respectively, and the TVC of both untreated and treated beef was augmented significantly with increasing storage time (0–12 d) (*p* < 0.05). It has been proposed that the safety limit for bacterial numbers in cooked meat products is <5 log CFU/g [31]. The count for untreated braised beef was 5.06 log CFU/g after 6 d, while BEO + GEO-treated beef had a 5.08 log CFU/g after 12 d. The results indicated that the combination of BEO and GEO has great potential to inhibit the reproduction of bacteria and extend the shelf life of braised beef. Stojanovic-Radic et al. [21] also observed similar results. The authors found that the number of *Salmonella* in chicken samples treated with BEO + rosemary EO decreased by 1.75 log CFU/g compared with the control group, indicating that BEO + rosemary EO showed an effective inhibitory effect on bacterial growth in chicken.

#### 3.3.2. pH Changes

As a critical parameter, pH can serve as an effective indicator of meat freshness. The pH changes in braised beef treated with BEO (1/2 MIC) + GEO (1/4 MIC) during storage at 4 °C are depicted in Figure 5B. During the initial period of storage (0–2 d), the pH of both untreated and EO-treated groups decreased (*p* < 0.05), which might be associated with the growth and reproduction of lactic acid bacteria (LAB). During the storage period, the initial pH of meat products will not hinder the growth of microbial cells, then a progressive acidification might occur (pH change from 6.0–6.5 to 5.0–5.3) owing to the fermentation of LAB [32]. With the increase in storage time (2–12 d), the pH of the beef samples exhibited fluctuations. The increase in pH might have been due to alkaline substances (such as amines and ammonia) degraded from protein and decarboxylation of amino acids catalyzed by microbial enzymes [33]. However, the change ranges of EO-treated beef were smaller than those of the control group, indicating that BEO + GEO helped to keep the pH stable. Behbahani et al. [25] also observed that the combination of *Satureja intermedia* and *Ducrosia anethifolia* EOs reduced pH change in minced beef compared to a control during storage.

#### 3.3.3. Changes in Texture Profile Analysis

Texture profile is also an important indicator for evaluating the quality and consumer acceptance of meat products. The texture profiles (hardness, resilience, and cohesiveness) of braised beef treated with GEO + BEO during storage are shown in Table 1. Hardness serves as the critical determinant of sensory perception, significantly impacting associated mechanical properties, including chewiness [34]. The hardness of both BEO + GEO-treated and untreated beef declined with the increase in storage time (0–12 d) at 4 °C (*p* < 0.05), which might have resulted from the structure of beef progressively loosening and the integrity of muscle tissue being compromised as the storage duration increased [35]. However, the hardness of the BEO + GEO treatments was higher than that of the control under the same storage time (*p* < 0.05). The antioxidant components in EOs could be beneficial for the maintenance of the texture of beef since the degree and distribution of fat in meat obviously affects the hardness. Moreover, the antioxidant activity of bioactive substances abundant in EOs could promote the generation of cross-links via Schiff base intermediates, inducing textural modification attributes on account of the carbonylation of proteins [36]. The resilience and cohesiveness of braised beef increased with increasing storage time, but there were no significant differences between the control and EO-treated samples (*p* > 0.05).

### 3.4. Color Changes

Visual appearance parameters, particularly surface color, significantly impact consumer acceptance with respect to meat products [37]. The color (L*, a*, and b*) changes in braised beef treated with the combination of GEO and BEO during storage are illustrated in Table 2. The L* value reflects meat brightness. The initial lower L* value in the treatment group could be attributed to the interaction between myoglobin and bioactive compounds of EOs [38]. During storage (0–12 d), L* values of both EO-treated and untreated beef increased, with a faster increase exhibited in the treated group than in the control. This might have been due to the loss of myoglobin because of the application of EOs on beef [39]. The a* value, an indicator of red color intensity, suggests less redness with lower values [40]. The initial a* values for the treatment and control groups were 5.84 and 6.73, respectively, indicating that the addition of EOs negatively affected the redness of braised beef. For the control group, a* values showed a decrease from 0 to 4 d, while a significant increase was observed at the end of storage (12 d) (*p* < 0.05). The reduction in a* values from 0 to 4 d might be attributed to lipid oxidation, which could have degraded the overall color of the meat products [41]. The increase at the end of storage might have been due to the impacts of microorganisms since it has been reported that *Macrococcus* could improve the color of food products [42]. As for the BEO + GEO group, the a* values at the end of storage (12 d) and at day 0 did not show a significant difference (*p* > 0.05). The antioxidant and antibacterial effects of BEO and GEO might explain the results. In terms of b* values, which serve as a quantitative indicator of yellowness intensity, elevated levels correlate with greater severity of microbial infection and enhanced lipid peroxidation [40]. The b* values of the BEO + GEO treatment and untreated beef samples were 13.59 and 13.81, respectively, on day 12. The BEO + GEO treatment showed a smaller increase in b* values, which might be attributed to the sustained antibacterial activity and antioxidant capacity of EOs effectively suppressing both microbial proliferation and lipid oxidation in the braised beef. Δ*E* can reflect the total color variation in meat products. Although the Δ*E* values of the BEO + GEO treatment were higher than those of the control during storage in this study, there were significant differences in the initial color parameters between the two groups. Moreover, the color changes in meat products during storage are affected by many factors, including lipid oxidation, microbial growth, and natural extracts.

## 4. Conclusions

The synergistic antibacterial effects of a combined BEO and GEO treatment on the preservation of braised beef were investigated in this study. BEO and GEO showed remarkable bacteriostatic effects on *E. coli* and *S. aureus* individually, and the combination of BEO and GEO had synergistic antibacterial effects, with an FICI of 0.75. BEO and GEO alone could decrease bacterial metabolic activity, reduce membrane potential, and destruct the intracellular enzyme and membrane integrity of *E. coli* and *S. aureus*. Moreover, the destructive effects of the combined BEO and GEO treatment were greater than single form. The application of combined BEO and GEO in braised beef effectively maintained the quality of beef by inhibiting the growth of bacteria, preventing texture change and color deterioration, and extending its shelf life by 6 d. This study revealed the synergistic antibacterial effect and mechanism of BEO and GEO, offering a promising strategy to enhance food preservation by prolonging product shelf life. However, the sensory properties and the stability of EOs within the food during storage were not investigated in this study. Therefore, future studies are expected to evaluate the sensory attributes and the stability of EOs in various food systems.

## Figures and Tables

**Table 1:** The changes in texture profiles of braised beef during storage at 4 °C.

Time (d)	Hardness (g)		Resilience		Cohesiveness	
	Control	BEO (1/2 MIC) + GEO (1/4 MIC)	Control	BEO (1/2 MIC) + GEO (1/4 MIC)	Control	BEO (1/2 MIC) + GEO (1/4 MIC)
0	14,548 ± 1102 ^aA^	11,136 ± 921 ^bA^	0.24 ± 0.01 ^aA^	0.22 ± 0.01 ^aA^	0.59 ± 0.03 ^aA^	0.55 ± 0.02 ^aA^
2	10,413 ± 1558 ^aB^	8198 ± 208 ^bB^	0.30 ± 0.04 ^aB^	0.28 ± 0.02 ^aB^	0.67 ± 0.03 ^aB^	0.64 ± 0.02 ^aB^
4	9997 ± 143 ^aB^	7402 ± 431 ^bC^	0.35 ± 0.02 ^aC^	0.29 ± 0.01 ^bB^	0.73 ± 0.02 ^aC^	0.65 ± 0.01 ^bB^
6	6766 ± 214 ^aC^	7273 ± 161 ^aC^	0.35 ± 0.03 ^aC^	0.31 ± 0.01 ^bB^	0.76 ± 0.04 ^aC^	0.66 ± 0.02 ^bB^
8	4677 ± 269 ^aD^	5392 ± 127 ^aD^	0.38 ± 0.01 ^aCD^	0.37 ± 0.01 ^aC^	0.77 ± 0.02 ^aC^	0.66 ± 0.01 ^bB^
10	4427 ± 97 ^aD^	4787 ± 139 ^aDE^	0.42 ± 0.01 ^aDE^	0.46 ± 0.03 ^bD^	0.86 ± 0.04 ^aD^	0.87 ± 0.03 ^aC^
12	3392 ± 380 ^aD^	4405 ± 228 ^aE^	0.46 ± 0.02 ^aE^	0.47 ± 0.03 ^aD^	0.63 ± 0.04 ^aAB^	0.53 ± 0.03 ^bA^

Note: Different capital letters (A–E) in the same column indicate significant differences within different times (*p* < 0.05). Different small letters (a,b) in the same row indicate significant differences within different treatment groups (*p* < 0.05).

**Table 2:** Color changes in braised beef during storage at 4 °C.

Time (d)	L*		a*		b*		Δ*E*	
	Control	BEO (1/2 MIC) + GEO (1/4 MIC)	Control	BEO (1/2 MIC) + GEO (1/4 MIC)	Control	BEO (1/2 MIC) + GEO (1/4 MIC)	Control	BEO (1/2 MIC) + GEO (1/4 MIC)
0	35.82 ± 0.51 ^aA^	33.81 ± 0.29 ^bA^	6.73 ± 0.16 ^aA^	5.84 ± 0.08 ^bABC^	13.37 ± 0.23 ^aAB^	11.58 ± 0.06 ^bA^	-	-
2	37.21 ± 0.39 ^aB^	35.46 ± 0.22 ^bB^	6.23 ± 0.05 ^aB^	6.31 ± 0.01 ^aCD^	13.59 ± 0.30 ^aAB^	12.78 ± 0.12 ^bBC^	1.49 ± 0.24 ^aA^	0.96 ± 0.11 ^bA^
4	37.32 ± 0.03 ^aB^	35.70 ± 0.31 ^bB^	5.43 ± 0.06 ^aD^	6.05 ± 0.02 ^bABCD^	13.14 ± 0.37 ^aBC^	12.54 ± 0.18 ^bB^	2.10 ± 0.08 ^aB^	1.33 ± 0.11 ^bA^
6	37.38 ± 0.37 ^aB^	36.92 ± 0.28 ^aC^	5.54 ± 0.16 ^aD^	6.15 ± 0.10 ^bBCD^	12.39 ± 0.18 ^aD^	13.32 ± 0.11 ^bDE^	2.27 ± 0.28 ^aB^	1.27 ± 0.28 ^bA^
8	37.37 ± 0.25 ^aB^	37.80 ± 0.08 ^aD^	5.87 ± 0.12 ^aC^	6.48 ± 0.49 ^bD^	12.70 ± 0.06 ^aCD^	13.03 ± 0.20 ^aCD^	1.98 ± 0.18 ^aAB^	2.10 ± 0.03 ^aB^
10	37.52 ± 0.07 ^aB^	38.02 ± 0.36 ^aD^	6.22 ± 0.11 ^aB^	5.61 ± 0.22 ^bA^	13.12 ± 0.15 ^aBC^	13.02 ± 0.01 ^aCD^	1.84 ± 0.03 ^aAB^	2.44 ± 0.15 ^abB^
12	37.65 ± 0.33 ^aB^	39.72 ± 0.09 ^bE^	6.77 ± 0.02 ^aA^	5.71 ± 0.16 ^bAB^	13.81 ± 0.42 ^aA^	13.59 ± 0.24 ^aE^	1.98 ± 0.19 ^aAB^	4.01 ± 0.03 ^bC^

Note: Different capital letters (A–E) in the same column indicate significant differences within different times (*p* < 0.05). Different small letters (a,b) in the same row indicate significant differences within different treatment groups (*p* < 0.05).

## Data Availability

The original contributions presented in the study are included in the article, further inquiries can be directed to the corresponding author.
